# Psychosis in youth in Singapore: a case series

**DOI:** 10.1192/bjo.2021.342

**Published:** 2021-06-18

**Authors:** Pei Ling Lim, Roselyne Shirley, Pat Fong

**Affiliations:** 1 Tan Tock Seng Hospital; 2 KK Women's and Children's Hospital

## Abstract

**Objective:**

In this report, we present a case series of children with psychotic symptoms referred to a child consultation liaison psychiatric service within a tertiary paediatric hospital in Singapore. The purpose of this case series is to identify common symptoms at presentation, review the current practices in our hospital for investigation and treatment of first episode psychosis and short-term outcomes.

**Case report:**

We identified 9 cases over a 1 year period, for which 7 were seen whilst admitted to hospital and 2 in the outpatient clinic. There were 6 females and 5 males ranging in age from 11 to 16 years old. The commonest symptoms on presentation were perceptual disturbance (88%) most commonly auditory hallucinations and altered behaviour (55%). Of the 7 children admitted to hospital, all were seen by the neurology team prior to the request for a psychiatric opinion. All admitted patients had blood and radiological investigations carried out. Most of the children were started on a short course of antipsychotic medication with the majority continuing to attend follow-up outpatient.

**Discussion:**

Only 9 cases were identified in this case series over a 1 year period highlighting that psychosis is not a common presentation in the paediatric population. From the history alone, it can be challenging to distinguish between primary and secondary causes of psychosis. Acute onset of symptoms and the presence of other neurological signs should raise the suspicion of an underlying organic cause. Out of 9 cases, only 1 case was treated for a presumed organic aetiology, which is consistent with findings from other authors who only found underlying organic factors in 12.5% of cases.

In this case series, we also noted that 45% of cases reported having symptoms for over 1 year before seeking help. This is also seen in the adult population in Singapore. Stigma, denial and lack of information about psychosis may all contribute to delay in seeking help. Although prolonged duration of untreated psychosis has been shown to be associated with poor long-term outcome, we found in our case series that even patients who reported a long duration of symptoms still responded well to medication.

**Conclusion:**

There is room for collaboration with our neurology colleagues in the approach towards children with first presentation of psychosis, both in terms of investigations and management. Identifying reasons for disengagement from psychiatric care is an area for further investigations to improve outcomes in our patients.

